# Measurement and Analysis of the Vibration Responses of Piano Soundboards with Different Structures

**DOI:** 10.3390/ma17051004

**Published:** 2024-02-22

**Authors:** Lan He, Yuwei Liang, Liang Zhang, Jing Zhou, Ruofan Wang, Zhenbo Liu

**Affiliations:** Key Laboratory of Bio-Based Material Science and Technology of Ministry of Education, Northeast Forestry University, Harbin 150040, China; namehelan@nefu.edu.cn (L.H.); lyw0824@nefu.edu.cn (Y.L.); zhliang17@nefu.edu.cn (L.Z.); zj@nefu.edu.cn (J.Z.); gbr_fff@nefu.edu.cn (R.W.)

**Keywords:** piano soundboard, vibration of free plate, mode shape, acoustic vibration response

## Abstract

The effect of structure on the vibration response was explored for four piano soundboards with different but commonly adopted structures. The vibration response was obtained using the free-vibration method, and the values of the dynamic modulus of elasticity and dynamic shear modulus obtained using the free-vibration frequency method (*E*_F_ and *G*_F_) were compared with the dynamic modulus of elasticity obtained using the Euler beam method (*E*_E_) and dynamic shear modulus obtained using the free-plate torsional vibration method (*G*_T_), respectively. It was found that the soundboards with different structures had different vibration modes and that excitation at different locations highlighted different vibration modes. For all the soundboards analyzed, the *E*_E_ and *G*_T_ were higher than *E*_F_ and *G*_F_ by 2.2% and 24.3%, respectively. However, the trends of the results of these methods were the same. The four piano soundboards with different structures possessed varying dynamic moduli of elasticity and dynamic shear moduli. These rules are consistent with the grain directions of the soundboards and the anisotropy of the wood (the direction of the units of the soundboards). The results show that the vibration mode of the piano soundboard is complex. The dynamic elastic modulus of the soundboard can be calculated using the Euler beam method. The results provide a reference for studies on the vibration response, material selection, production technology, and testing of piano soundboards.

## 1. Introduction

The piano is widely appreciated for its delicate and expressive emotional range, broad tonal range, and beautiful timbre. The soundboard is a crucial component responsible for sound transmission and resonance in the precise and complex structure of the piano [1]. The piano soundboard is equipped with bridges and ribs, enabling the soundboard to connect to the strings and radiate sound into the air [2]. The soundboard, a large wood panel, determines the resonance effects, levels, and richness of overtones and propagation speed of the sound. Studying the properties of a soundboard is crucial to improving a piano’s timbre. Soundboards are made from wood. Achieving excellent tones and rich overtones requires a wood that propagates sound waves at high speeds, has good elasticity, is lightweight, and has low density. Coniferous woods, such as Western Canada spruce, fish scale spruce, and fir, offer good acoustic vibrations. There are sometimes strict requirements for the origin, sawn timber, moisture content, density, defects, and processing technology of the wood [3].

During play, the soundboard converts energy into sound through its structure and radiates the sound into the air. The structure of the soundboard is thus important to the acoustic quality of a piano. Currently, the two commonly used types of piano soundboards are boards made from solid wood and solid wood composite. A solid wood soundboard is fabricated by diagonally splicing radially cut plates that are approximately 10 cm wide, whereas a solid wood composite soundboard is fabricated by gluing face and core plates at a certain angle to form a three-layer splicing plate. The surface and core plates of the solid wood composite soundboard are fabricated by splicing radially cut plates of spruce species. Surface plates must be composed of good material, whereas core plates can have slightly reduced material standards. The solid wood composite soundboard is assembled by gluing together the core and surface plates, with their textures interlaced. The core plate can be assembled vertically, horizontally, or diagonally. The overall soundboard should be tightly and firmly assembled, without gaps, delamination, or other defects. The mechanical properties and acoustic vibration quality of the soundboard are affected by its structure, as the complex structure, large size, and wood anisotropy of the soundboard result in vibrations in multiple modes with varying frequencies. It has been noted that the vibration frequencies and modes differ between soundboards with different structures [4].

In terms of the material itself, scholars have analyzed several common influencing factors, such as tree species [5,6], geometric shape [7], moisture content, density, porosity, quality grade, etc. [8,9]. Of course, some scholars are seeking optimization methods for soundboards, such as through chemical impregnation [10] or by conducting finite element simulations on the soundboard manufacturing process to find optimization solutions [11]. Scholars have sought the acoustic effects and influencing factors of pianos through designed models [12,13,14,15]. Boutillon regarded different parts of the soundboard as basic structures, proposing a semi-analytical model [16]. In terms of method proposals and optimizations, many scholars have made corresponding efforts. Zhou J. calculated boundary conditions and precision [17]. Ege separated the nonlinearity of the resonance board from that of the excitation device and measured them [18]. Opazo-Vega proposed a non-destructive method for measuring local variations [19]. Ou D. proposed an inherent frequency optimization method for plate structures based on the finite element method and genetic algorithm [20]. Trévisan B. proposed a method for analyzing the acoustic vibration characteristics of orthotropic, ribbed, non-rectangular fixed plates [21]. Guan suggested that four-point support could be used for the elastic performance measurement of full-size wooden composite panels [22]. To visualize the vibration modes of soundboards, numerous explorations of modal shapes have also been undertaken. Yang X. used transfer functions to detect the bending vibration waveform of the first-order mode of a wooden beam [23]. Mania compared the modal parameters of resonant and non-resonant materials [24]. The piano soundboard usually has a large area, with a length of up to 2000 mm and a width of up to 1200 mm. The soundboard thickness typically ranges from 7 to 10 mm. To determine its properties, the soundboard is usually sawn into wooden strips and tested using the bending vibration method [25,26]. This method is straightforward and efficient to implement. However, when sawing the sample, it is necessary to consider the grain angle of the soundboard splicing so as to not damage the test piece. Concurrently, this method imposes specific requisites on the magnitude of the sample [27,28]. In addition, it is challenging to assess the effect of the grain angle on the acoustic vibration characteristics of the soundboard. Furthermore, the torsional vibration of the soundboard is often overlooked. Currently, the primary challenges in determining the performance of a soundboard of a piano are as follows. A soundboard’s vibration can be determined using the bending vibration method, through experimental modal determinations, and by adopting other practical methods. However, owing to the complexity of the vibrations, empirical judgment alone may result in frequency attribution errors. Computer simulations and other methods can also be adopted to obtain the soundboard vibration and response. However, soundboards with different structures are usually simplified in the modeling process. Numerical studies cannot use a block of wood to accurately explore the performance of an actual soundboard. Numerical and experimental results have been obtained for many wood composite panels [24,29,30,31,32]. Typically, the performance of a soundboard is obtained by solving the free-vibration frequency equation of a thin wooden plate.

This paper analyzes four piano soundboards with different structures according to the principle of the free vibration of a square plate. The vibration modes of the soundboards were measured in experiments to confirm the vibration frequencies. The dynamic modulus of elasticity (*E*_F_) and dynamic shear modulus (*G*_F_) were derived for each of the four soundboards using the free-vibration frequencies of thin wooden plates. Then, the obtained values *E*_F_ and *G*_F_ were compared with the *E*_E_ obtained using Euler’s free-beam method and *G*_T_ obtained using the method of the torsion of free plates. This study determined the (2,0)-, (0,2)-, (1,1)-, and (2,2)-order vibration modes of the four piano soundboards with different structures. It also compared different methods for calculating a specimen’s dynamic elastic modulus and shear modulus. This comparison enables the determination of the vibration response characteristics of large-scale soundboards and provides a reference for optimizing the manufacturing of piano soundboards.

## 2. Materials and Methods

### 2.1. Materials

**Figure 1:**
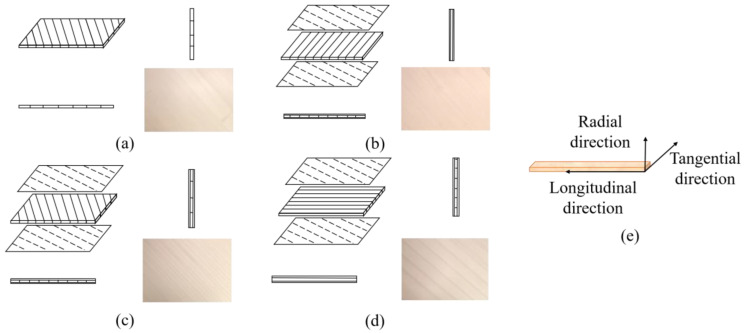
Four structures of piano soundboards: (**a**) tone-wood soundboard (A); (**b**) laminate soundboard with vertical core (B); (**c**) laminate soundboard with incline core (C); and (**d**) laminate soundboard with horizontal core (D); and (**e**) the wood direction of the units.

The present study was conducted for four upright piano soundboards that had different structures (i.e., different composite processes) and were produced by Chengdu CANYA Wood Industry Company, Ltd. (Chengdu, China). These 4 structures are the most commonly used soundboard structures in pianos currently. The soundboards were glued using urea–formaldehyde resin adhesive. The dimensions of the boards were half those of actual soundboards used in piano products for the purpose of easy measurements. The surface and core boards were composed of radially cut fish scale spruce. Figure 1 shows the structures of the four piano soundboards and the wood direction of the units, and Table 1 lists the composite types and parameters. The boards were placed in a room-temperature environment to achieve a moisture content of 7–9%, in accordance with standard requirements (QB/T 2978-2008 [33]).

### 2.2. Free-Plate Torsional Vibration Method

Each board was a rectangular thin plate with an aspect ratio of approximately 1.4, with its length and width dimensions being much larger than the thickness dimension. The length-to-thickness ratio was as high as 100.

**Figure 2:**
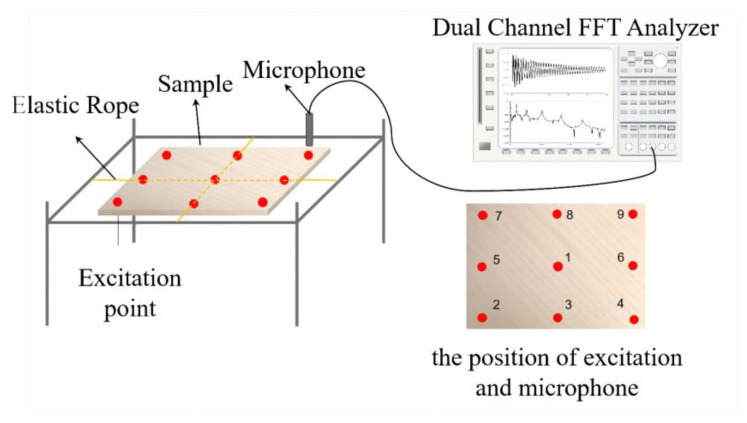
Measuring system with cross-suspension support and the positions of excitation and of the microphone.

Figure 2 shows the system used to measure the free vibration of the board and the location of the hammer excitation point. The board was suspended crosswise at the centerlines of its length and width using elastic rope. The positions of the microphone (ONO SOKKI, Kanagawa, Japan) and hammer (Dytran, Chatsworth, CA, USA) that produced desired vibration modes were determined. The board was excited by tapping the generated free vibrations. The collected signals were transmitted to a multi-channel fast Fourier transform analyzer (ONO SOKKI, Kanagawa, Japan), which produced time- and frequency-domain plots of the specimen’s vibration. The analyzer calculated the resonance frequency of each order.

To examine the effect of texture on the free-vibration determination, the four soundboards were tested with a diagonal setup, as described in the following. In the measurement process, the excitation and microphone positions were located at opposite ends of a diagonal line on the specimen, such as at the [4,7] and [2,9] positions. Here, the first number refers to the excitation position and the second number to the microphone position. The line between the excitation point and microphone was approximated to be in the direction of the smooth or transverse surface plate texture, as shown in Figure 2. The experiments maintained a consistent excitation strength and were repeated multiple times.

### 2.3. Vibration Frequencies of the Soundboards

**Figure 3:**
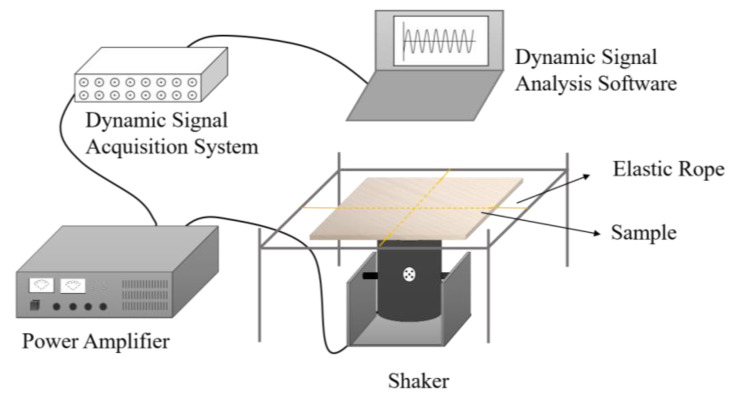
Measuring system for experimental modal determination.

The inherent anisotropy of the wood, coupled with the intricate architecture of the soundboard, renders the task of ascertaining the resonant frequencies associated with piano soundboards exhibiting diverse grain orientations a formidable challenge. Empirical judgement is difficult, and the experimental modal determination of the frequency corresponding to the vibration mode is thus necessary. Figure 3 shows the measurement system. The specimen was fixed using the cross-hanging method, with the center positioned directly above the shaker (ZOSON, Yangzhou, China), which was connected to a power amplifier (ZOSON, Yangzhou, China). A dynamic signal acquisition and analysis system V9.0 (ZOSON, Yangzhou, China) was used to input a sinusoidal wave signal, and the specimen was subjected to forced vibration caused by an exciter. Black quartz sand was sprinkled on the specimen to visualize its vibration geometry pattern through the formation of Chladni figures [34]. In conducting a frequency sweep, the vibration mode was observed at different frequencies.

### 2.4. Dynamic Modulus of Elasticity and Dynamic Shear Modulus

The vibration of a plate is complex. In adopting the Rayleigh method to solve the differential equation of the transverse free vibration of an orthotropic and anisotropic thin plate, the equation for the frequency of the free vibration of a wooden thin plate (Equation (1)) was used to calculate the dynamic modulus of elasticity and dynamic shear modulus. This requires the (2,0)-, (0,2)-, (1,1)-, and (2,2)-order frequencies of the vibration of the thin plate (Equations (2)–(4)) [35]. If we calculate the dynamic modulus of elasticity (*E*_E_) of the specimen using the Eulerian free-beam method [36] and determine the shear modulus (*G*_T_) using the free-plate torsional vibration method [30], we need only the first-order bending and first-order torsional frequencies of the specimen vibration (Equations (5)–(8)), which are the frequencies of (2,0) and (1,1) orders.

(1) $$f_{\left(m,n\right)}=\frac{1}{2\pi }\sqrt{\frac{D_{11}\frac{\alpha_{1(m,n)}}{a^{4}}+D_{22}\frac{\alpha_{2(m,n)}}{b^{4}}+2D_{12}\frac{\alpha_{3(m,n)}}{a^{2}b^{2}}+4D_{66}\frac{\alpha_{4(m,n)}}{a^{2}b^{2}}}{\rho h}}$$

where *D*_11_ and *D*_22_ are the bending stiffnesses of the thin plate in the elastic principal direction, and *D*_66_ is the torsional stiffness of the thin plate in the elastic principal direction. *a*, *b*, and *h* are, respectively, the length, width, and thickness of the specimen in meters, and *ρ* is the density in kg·m^−3^.

The resonance frequency of an (m,n) order is denoted by *f*_(m,n)_. The constant coefficients *α*_1(m,n)_, *α*_2(m,n)_, *α*_3(m,n)_, and *α*_4(m,n)_ are obtained from Table 2 [35].

Equation (1) has been solved and simplified in the literature. The dynamic modulus of elasticity and dynamic shear modulus have been calculated using Equations (2)–(4):

(2) $$E_{xF}=\frac{12}{h^{3}}D_{11}\left(1-\mu_{x}\mu_{y}\right)$$

(3) $$E_{yF}=\frac{12}{h^{3}}D_{22}\left(1-\mu_{x}\mu_{y}\right)$$

(4) $$G_{F}=\frac{12}{h^{3}}D_{66}$$

where *E*_F_ is the dynamic modulus of elasticity in Pascals, *G*_F_ is the dynamic shear modulus in Pascals, and (1 − *μ_x_μ_y_*) is typically assumed to be 0.99 [7].

Equation (5) was used to calculate the dynamic modulus of elasticity (*E*_E_) using the Eulerian free-beam method:

(5) $$E_{E}=\frac{48\pi^{2}\rho a^{4}f_{b}^{2}}{\beta^{4}h^{2}}$$

where *a*, *b*, and *h* are, respectively, the length, width, and thickness of the specimen in meters. The density of the specimen is denoted by *ρ* in kg/m^3^. The constant *β*, which relates to the boundary conditions, is taken as 4.73. The first-order bending frequency of the free-plate vibration is denoted by *f*_b_ in Hertz.

Equation (6) was used to calculate the shape factor (*β*) of the free-plate torsional vibration law for a rectangular cross-section:

(6) $$\beta =\frac{1}{16}\left[\frac{16}{3}-3.36\frac{h}{a}\left(1-\frac{h^{4}}{12a^{4}}\right)\right]$$

Equation (7) was used to calculate the dynamic shear modulus (*G*_T_) of a square solid-wood plate:

(7) $$G_{T}=\frac{\pi^{2}\rho \left(\frac{abf_{t}^{2}}{h}\right)}{32.48\beta }$$

Equation (8) was used to calculate the dynamic shear modulus (*G*_T_) of a square solid wood composite plate:

(8) $$G_{T}=\frac{\pi^{2}\rho \left(\frac{abf_{t}^{2}}{h}\right)}{30.87\beta }$$

where *a*, *b*, and *h* are, respectively, the length, width, and thickness of the specimen in meters, *ρ* is the density in kg·m^−3^, and *f*_t_ is the first-order torsional frequency of free-plate vibration in Hertz.

In the present experiment, specimen A was a solid wood soundboard, for which Equation (7) was applied, whereas specimens B, C, and D were laminate soundboards, for which Equation (8) was applied.

## 3. Results and Discussion

### 3.1. Multi-Point Excitation Test

**Figure 4:**
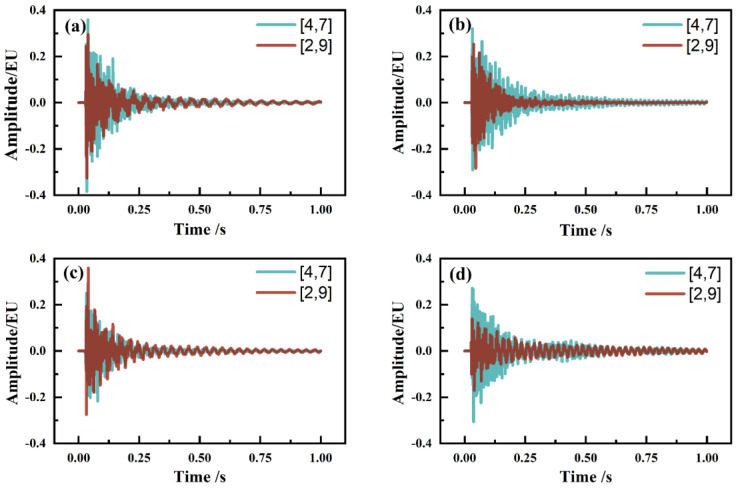
Time domains of four kinds of piano soundboards measured at locations [4,7] and [2,9]: (**a**) tone-wood soundboard (A); (**b**) laminate soundboard with vertical core (B); (**c**) laminate soundboard with incline core (C); and (**d**) laminate soundboard with horizontal core (D).

**Figure 5:**
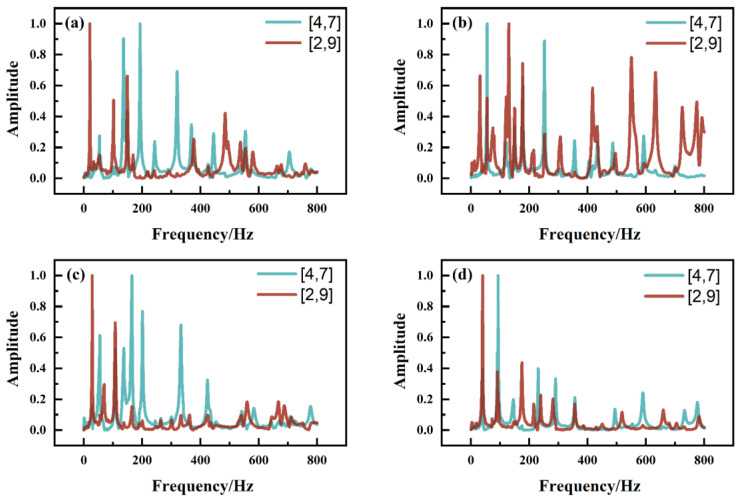
Frequency domains of four kinds of piano soundboards measured at locations [4,7] and [2,9]: (**a**) tone-wood soundboard (A); (**b**) laminate soundboard with vertical core (B); (**c**) laminate soundboard with incline core (C); and (**d**) laminate soundboard with horizontal core (D).

To accurately determine the vibration frequency of each order of the piano soundboard and to investigate the effect of the grain direction of the surface plates on the vibration responses of the soundboards, each sample was tested through multi-point excitation. It was found that different excitation points highlighted different vibration modes of the soundboard, e.g., the (2,0), (0,2), and (1,1) vibration orders were highlighted when the excitation point and microphone position were on a diagonal line. The frequency-domain data obtained for the excitation of the four soundboards at the [4,7] and [2,9] point positions were normalized separately, and different time-domain and frequency-domain plots were obtained (Figure 4 and Figure 5). The values of the resonance frequencies and amplitudes are listed in Table 3.

In the [4,7] and [2,9] point excitation tests, the main frequencies in the frequency-domain graphs of a soundboard were basically the same, whereas the maximum difference in the excitation frequency of the same order frequency at different points was 6.4 Hz. Regardless of the point position, the acoustic wave vibrated throughout the plate and was received by the microphone, and the whole-plate vibration frequency was thus basically the same. However, the difference in the frequency-domain vibration amplitude was larger between [4,7] and [2,9] point excitation tests, i.e., during the (2,0)-order vibration of sample A, the [2,9] point excitation amplitude was approximately 13 times the [4,7] point excitation amplitude (Table 3). This result is due to the different excitation points highlighting different types of vibration of the soundboard, with the vibration type corresponding to the frequency of the largest vibration amplitude. The first-order torsional frequency, namely the (1,1)-order frequency, was more prominent in the frequency-domain plot obtained for the [4,7] point excitation, whereas the first-order bending frequency, namely the (2,0)- and (0,2)-order frequencies, was more prominent in the frequency-domain plot obtained for the [2,9] point excitation. Therefore, to obtain the first-order bending frequencies ((2,0)- and (0,2)-order frequencies), measurements can be made parallel to the grain direction of the panel. To obtain the first-order torsional frequency ((1,1)-order frequency), measurements can be made perpendicular to the grain direction of the panel. In future research on piano soundboards, we will use this result to judge the vibration frequency of a sample belonging to a vibration type and explore a more convenient and faster method of determining the vibration response of a piano soundboard. We will also explore the effects of the soundboard texture angle and the string angle on the piano sound when the piano soundboard is assembled.

### 3.2. Vibration Frequencies of the Four Piano Soundboards with Different Structures

Soundboards undergo complex vibrations, as the frequencies of different orders are similar. The selection of the frequencies of orders (2,0), (1,1), (0,2), and (2,2) of the soundboards had a direct effect on the accuracies of the dynamic elastic modulus and dynamic shear modulus. It is thus necessary to accurately determine the vibration frequencies when conducting vibration characteristic tests. In the present experiment, each soundboard was excited at multiple points one by one to identify each order of the vibration frequency. In the spectrogram of the free-plate specimen (Figure 5), the (2,0)-, (1,1)-, (0,2)-, and (2,2)-order frequencies generally corresponded to the first four peaks, and the exact order relates to the dimensions of the specimen and the way that the specimen is bonded. To ensure the correct frequency assignment, the frequencies corresponding to the first four peaks in the free-vibration frequency-domain diagram of the specimen were verified through experimental modes. The vibration modes of the first four frequency orders of the four soundboard specimens were derived.

**Figure 6:**
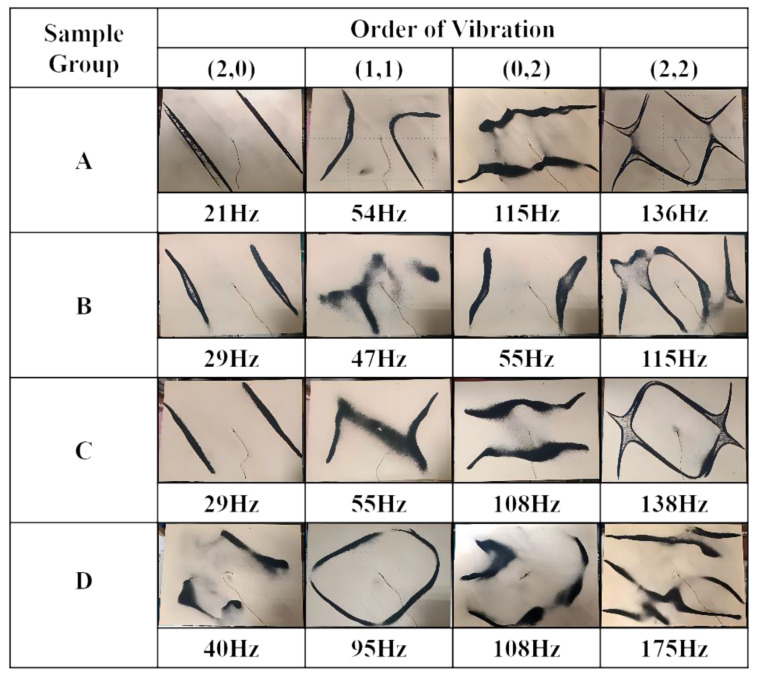
Modal shapes of orders (2,0), (1,1), (0,2), and (2,2) of four kinds of piano soundboards.

The test results reveal that the directions of the nodal lines of the soundboards were affected by the anisotropy of the wood and the structure of the soundboards. In the case of an isotropic square plate under free vibration, the nodal line was usually axisymmetric relative to the line connecting the midpoints of the opposite edges of the axis [36]. In Figure 6, the direction of the nodal line of the soundboard is obviously not axisymmetric with the midpoint of the opposite edge but rather lies at an angle to the length and width directions of the specimen. The nodal line direction of the solid wood soundboard was in the longitudinal direction of the spliced lath, and the nodal line direction of the laminated soundboard was approximately the longitudinal direction of the surface grain of the panel. The vibration mode of the tone-wood soundboard and that of the laminate soundboard with an inclined core were clearer and similar to that of the others. The vibration mode of the laminate soundboard with a vertical core and that of the laminate soundboard with a horizontal core were more special. Because regardless of the direction of the wood core board, the (2,0) modal shape of the three kinds of laminated boards approximated the direction of the panel texture. The surface plate grain directions of the soundboards had a great influence on the angles of the mode shapes. As two (1,1) vibration modes of the laminate soundboard with a vertical core and of the laminate soundboard with a horizontal core can be inferred, in the case of free-plate vibration, the surface plate grain direction of the sample vibration mode direction has a greater impact on the angle of the shapes of the vibration mode, and the core plate splicing angle will have a greater impact on the shape of the vibration mode. The mode shapes of the four soundboards with different structures were not the same. The vibration mode of the tone-wood soundboard was more regular than those of the laminate soundboards. The texture angles of the laminate soundboards were more complex, and the vibration mode regularity and symmetry were weaker than those of the tone-wood soundboard. The nodal lines of thee four soundboards with different structures were not smooth, and the modes were not regular or symmetrical like that of an isotropic square plate [34]. It is relatively difficult to judge which order the frequency belongs to according to the mode shape.

### 3.3. Comparison of Results Obtained Using the Free-Vibration Frequency Method for Thin Wooden Plates and Those Obtained Using the Eulerian Free-Beam Method and the Free-Plate Torsional Vibration Method

**Figure 7:**
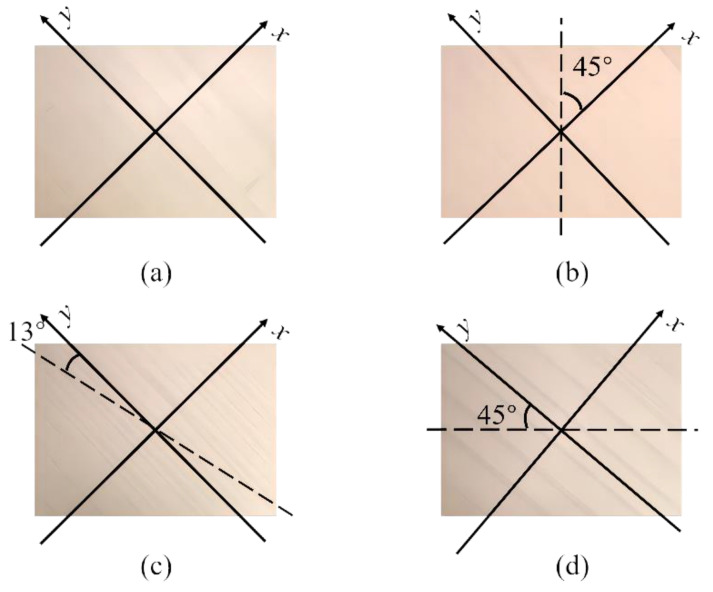
Axis setting and the orientation of surface layers and the cores of the piano soundboards (the dashed lines indicate the directions of the core): (**a**) tone-wood soundboard (A); (**b**) laminate soundboard with vertical core (B); (**c**) laminate soundboard with incline core (C); and (**d**) laminate soundboard with horizontal core (D).

As the surface plate texture affects the direction of the soundboard mode shapes, the lines of the mode shapes were not aligned with the length or width of the soundboard but were at a certain angle of inclination. Therefore, the results based on the vibration corresponding frequency of the vibration mode should be the dynamic modulus of elasticity in line with the direction of the vibration mode of the soundboard. This result is consistent with the structure of the soundboard and the characteristics of anisotropic wood within a certain degree of error. Using the vibration mode of the soundboard to determine the *x* and *y* coordinate axes (Figure 7). The frequencies of the (2.0), (1.1), (0.2), and (2.2) orders were input into Equations (2)–(4), using the free-vibration frequency of the thin wooden plate method for the calculation to obtain *E*_F_ and *G*_F_. And *E*_F_ and *G*_F_ were compared with the results of *E*_E_, which was obtained using the Euler free-beam method, and *G*_T_ was obtained using the free-plate torsional vibration method (Table 4). We explored a simpler and faster determination of the parameters for the calculation of the soundboard vibration.

The analysis of the calculations made using the free-vibration frequency method for thin wooden plates shows that in the *x*-direction, dynamic Young’s modulus was largest for specimen D (2.308 GPa), followed by specimen B (1.588 GPa) and specimen A (0.501 GPa), whereas in the *y*-direction, dynamic Young’s modulus was largest for specimen C (4.702 GPa), followed by specimen A (3.796 GPa) and specimen B (1.351 GPa). These results mainly relate to the grain directions of the surface and core plates of the soundboard. For specimens A and C, the *x*-direction is equivalent to the radial direction of the wood and has a smaller modulus of elasticity, whereas the *y*-direction is equivalent to the longitudinal direction of the wood and has a larger modulus of elasticity. For specimens B and D, the *x*- and *y*-directions are the directions of the angle between the longitudinal and radial directions of the wood plates that make up the soundboards, and the difference in the moduli of elasticity between the *x*- and *y*-directions is thus small. The dynamic shear modulus is largest for specimen D (5.457 GPa), followed by specimen C (2.040 GPa) and specimen B (1.575 GPa). Again, these results relate to the grain angle of the constituent units of the soundboards. Sound waves in the laminate soundboard with a horizontal core propagate along the length direction of the core plate unit, for which there are fewer inter-unit bonding parts. In the laminate soundboard with a vertical core, sound waves propagate along the width direction of the core plate unit, for which there are more bonding parts. The glued part of the laminate soundboard with an inclined core and that of the tone-wood soundboard are basically the same. However, the laminate soundboard with an inclined core is thinner than the tone-wood soundboard, and both have a top and bottom layer of thin plate. The laminate soundboard with an inclined core was more resistant than the tone-wood soundboard to torsional deformation [37,38], and sandwich boards can improve the acoustic properties of the soundboard [39].

Table 3 shows that the dynamic moduli of elasticity and dynamic shear moduli of the four soundboards obtained using the free-vibration differential equation for a thin wooden plate are consistent with those obtained using the Euler free-beam method and free-plate torsional vibration method. The results obtained using the free-vibration frequency method for a thin wooden plate are less than those obtained using the Euler free-beam method and free-plate torsional vibration method. Among the results, the dynamic moduli of elasticity obtained using the free-vibration frequency method and the Euler free-beam method are similar, and the result obtained using the Euler free-beam method is approximately 2.2% higher than that obtained using the free-vibration frequency method for a thin wooden plate. The dynamic shear modulus calculated using the free-vibration frequency method for a thin wooden plate is approximately 24.3% higher than that obtained using the free-plate torsional vibration method. It appears that the discrepancies observed may stem from the specimen size not strictly adhering to the specifications of the Euler–Bernoulli beam theory, leading to potential inaccuracies. Additionally, there is a discernible disparity between the actual structure of the specimen and the idealized structure assumed in the derivation of the free torsion method, which could further contribute to these differences. In future research, we plan to correct the formula for the angle of the soundboard splice to obtain a formula that is more practical for sandwich composites with an angle. In a practical determination of soundboard properties, such as a quick and simple determination for judging the nature or merit of a soundboard in a factory, the Euler free-beam method can be used to calculate the dynamic modulus of elasticity and the free-plate torsion method can be used to calculate the dynamic shear modulus.

## 4. Conclusions

This study adopted the square-plate free-vibration method to analyze four piano soundboards with different structures. The mechanical properties of the four soundboards were compared by determining the vibration frequencies of the soundboards experimentally. In addition, *E*_F_ and *G*_F_ obtained using the free-vibration frequency method for thin wooden plates were compared with *E*_E_ obtained using the Euler free-beam method and *G*_T_ obtained using the free-plate torsion method. The following conclusions were drawn from the results of the study:

(1) Excitations at different points highlight the different modes of vibration of the soundboards. To obtain the (2,0)- and (0,2)-order frequencies, measurements can be made parallel to the grain direction of the panel. To obtain the (1,1)-order frequency, measurements can be made perpendicular to the grain direction of the panel.
  Different piano soundboards can be spliced differently. Furthermore, splicing results in complex texture angles, and in vibration modes that are less regular and symmetric than those of isotropic materials. It is thus more difficult to judge the vibration mode of a spliced soundboard.
(2) *E*_F_ and *G*_F_ calculated using the free-vibration frequency method for a thin wooden plate are similar to *E*_E_ calculated using the Euler free-beam method and *G*_T_ calculated using the free-plate torsional vibration method. The obtained trends are the same. In the *x*-direction, the resistance to bending deformation decreases in the order of *E_x_*_D_ > *E_x_*_B_ > *E_x_*_C_ > *E_X_*_a_. In the *y*-direction, the order is *E_y_*_C_ > *E_y_*_A_ > *E_y_*_D_ > *E_y_*_B_. The resistance to torsional deformation decreases in the order of *G*_D_ > *G*_C_ > *G*_A_ > *G*_B_. In practical applications, Euler’s free-beam method and the free-plate torsional vibration method can be used to conduct simple calculations for comparing and judging the mechanical properties and vibration characteristics of different soundboards.
(3) This investigation delved into the impact of various soundboard structures on the vibrational responses and modal patterns of piano soundboards. The study has measurement orientations conductive to the expedited acquisition of (2,0), (0,2), and (1,1) modal frequencies. Furthermore, it deduced the elastic and shear moduli for rectangular soundboards, which can be conveniently computed using Euler–Bernoulli beam theory and the free-torsion method. Prospective research avenues may encompass examining the influence of core board angles on the soundboard’s vibrational dynamics, as well as making enhancements to the free-torsion method.

## Figures and Tables

**Table 1:** Composite conditions and basic parameters of piano soundboards.

Sample Group	Type	Core	Angles of the Surface Plate to the Cores	Dimensions (mm)	Density (kg·m^−3^)
A	tone-wood soundboard	—	47°	698.86 × 497.91 × 9.26	481.05
B	laminate soundboard	vertical core	90°/45°	698.26 × 498.53 × 7.37	443.85
C	laminate soundboard	incline core	55°/42°	698.51 × 498.22 × 8.21	447.79
D	laminate soundboard	horizontal core	180°/45°	699.20 × 496.23 × 7.98	415.53

**Table 2:** Constant coefficient *α*_(m,n)_.

(m,n)	*α*_1_	*α*_2_	*α*_3_	*α*_4_
(1,1)	0	0	0	144
(0,2)	0	500.6	0	0
(2,0)	500.6	0	0	0
(2,2)	500.6	500.6	151.3	2448.3

**Table 3:** The values of the resonance frequencies and amplitudes.

Sample Group	Excitation Point	Order of Vibration							
		(2,0)		(1,1)		(0,2)		(2,2)	
		*f*/Hz	*A*	*f*/Hz	*A*	*f*/Hz	*A*	*f*/Hz	*A*
A	[2,9]	20	0.543	55.3	0.102	105.3	0.143	134.8	0.057
	[4,7]	20	0.042	53	0.201	111.7	0.027	134.9	0.651
B	[2,9]	29.5	0.158	46.4	0.045	53.2	0.109	116.6	0.137
	[4,7]	30.5	0.514	42.125	0.061	55.375	1.000	118.625	0.222
C	[2,9]	29	0.583	55.5	0.049	110.1	0.342	139.1	0.036
	[4,7]	29	0.136	55.2	0.485	110.6	0.146	138.9	0.331
D	[2,9]	40	1.000	91	0.271	108	0.027	174.8	0.437
	[4,7]	40	0.394	94	0.475	108	0.061	174.6	0.029

Note: *f* is the frequency, and *A* is the normalized amplitude.

**Table 4:** Comparison of the results of the free-vibration frequency method of thin wooden boards with the Euler beam and free-plate torsional vibration methods.

Sample Group	*E_x_* (GPa)		Δ*E_x_* (GPa)	*E_y_* (GPa)		Δ*E_y_* (GPa)	*G* (GPa)		Δ*G* (GPa)
	*E_xF_*	*E_xE_*		*E_yF_*	*E_yE_*		*G_F_*	*G_T_*	
A	0.501 (8.7%)	0.507 (8.7%)	−0.006	3.797 (12.6%)	3.839 (12.6%)	−0.043	1.618 (9.3%)	1.976 (9.3%)	−0.357
B	1.588 (3.6%)	1.606 (3.6%)	−0.023	1.351 (7.5%)	1.369 (10.2%)	−0.018	1.575 (12.3%)	1.917 (12.3%)	−0.342
C	1.169 (5.8%)	1.268 (12.7%)	−0.013	4.702 (11.3%)	5.141 (18.6%)	−0.044	2.040 (10.9%)	2.680 (17.8%)	−0.64
D	2.309 (0.6%)	2.335 (0.6%)	−0.026	1.869 (1.4%)	1.890 (1.4%)	−0.021	5.457 (4.2%)	6.655 (4.3%)	−1.198

Notes: Δ*E_x_* and Δ*E_y_* are the difference between the free-vibration frequency method of thin wooden boards and the Euler beam method. Δ*G* is the difference between the free-vibration frequency method of thin wooden boards and free-plate torsional vibration method. The coefficients of variation are in parentheses.

## Data Availability

Data are contained within the article.
